# Rubber voice illusion exposed neural correlates of voice perception and vocal adaptation across the continuum of psychosis

**DOI:** 10.1038/s41598-025-34714-9

**Published:** 2026-01-08

**Authors:** Suong Welp, Andrea Hildelbrandt, David A. Magezi, Martin Voss, Laura Kaltwasser

**Affiliations:** 1 Department of Psychology, Carl von Ossietzky Universität Oldenburg, 26129 Oldenburg, Germany; 2 Max Planck Institute for Human Cognitive and Brain Sciences, 04103 Leipzig, Germany; 3 Cluster of Excellence Hearing4all, Carl von Ossietzky Universität Oldenburg, Oldenburg, Germany; 4 Biological Psychology and Neuropsychology, University of Hamburg, 20146 Hamburg, Germany; 5 Department of Psychiatry and Psychotherapy (Charité Campus Mitte), Charité University Medicine and St. Hedwig Hospital, 10115 Berlin, Germany; 6 Berlin School of Mind and Brain, Humboldt-Universität zu Berlin, 10117 Berlin, Germany

**Keywords:** Auditory illusion, Self-perception, Pitch adaptation, Speaking-induced suppression, Schizotypy, Predictive coding, Neuroscience, Psychology, Psychology

## Abstract

The Rubber Voice Illusion is an auditory analogue of the classic Rubber Hand Illusion, a “mind-trick” for the voice. When speakers hear a stranger’s utterances that are perfectly time‑locked and syllable‑matched to their own voice, many momentarily adopt that alien voice as self‑generated. We used this illusion to probe the integrity of predictive auditory mechanisms across the psychosis continuum. Sixty healthy adults completed three Talk‑Listen conditions while high‑density EEG was recorded: veridical (live playback of own voice), stranger‑match (congruent stranger voice) and stranger‑mismatch (incongruent). Behaviorally, only the congruent condition evoked a sense of Ownership and Agency over the stranger’s voice, paired with an upward shift in fundamental frequency toward the higher-pitched stranger voice - an implicit indication of voice adaptation. Neurophysiologically, the canonical speaking-induced suppression was replicated, and suppression was significantly smaller in participants who scored high on Peters et al. Delusions Inventory. In controls, greater pitch shift was associated with stronger neural suppression, suggesting a functioning self-suppression mechanism; conversely, in high-schizotypy individuals, the relationship reversed, despite pitch shifting toward the stranger’s pitch, they exhibited reduced suppression, indicating decoupling between motor output and sensory prediction. The study provides further evidence to the intricate relationship of neural mechanisms underlying self-generated actions with sensory processing in the psychosis continuum.

**Supplementary Information:**

The online version contains supplementary material available at 10.1038/s41598-025-34714-9.

## Introduction

Research on how the brain keeps the *self* separate from the *world* has long relied on illusions or “mind-trick” paradigms that deliberately blur that boundary. Voluntary action ordinarily fuses two minimal-self signals. Sense of Ownership (SO) tags incoming sensory signals as belonging to *me*, whereas sense of Agency (SA) tags events as *caused by* me^1^. In everyday behaviors SO and SA are fused, yet they can be teased apart by illusions or by certain psychiatric symptoms such as thought insertion and auditory hallucinations.

Classic demonstrations like the Rubber Hand Illusion show that synchronizing vision and touch can make people adopt a plastic hand as their own^2^. This principle generalizes to the face^3^, the whole body^4^ and, crucially for the present work, the voice^5^. Because speech is the channel through which auditory-verbal hallucinations manifest, voice-ownership illusions provide a uniquely relevant probe of SO and SA in the auditory domain. In the *Rubber Voice Illusion*^5^ (RVI), by making the auditory feedback congruent (temporally and phonetically) with the participant’s own vocalization, they successfully induced a SO over the stranger’s voice (stranger’s voice perceived as a modified version of participant’s own voice). When the auditory feedback was congruent with one’s own vocalization (phonetic content, onset and offset), the RVI took place. When the linguistic congruence was broken, the illusion was abolished. Zheng and colleagues^5^ proposed that phonemically incongruent, but temporally aligned, auditory feedback violates the self-monitoring system of the body, terminating the perceived voice-ownership. These studies have demonstrated how malleable our body perception is, and how tightly it is connected to self-identification. One short exposure to the manipulated congruent multisensory feedback would let us adopt a stranger’s physical trait as our own and subsequently alter our self-perception.

For most people the *self* feels automatic and stable; for patients with certain psychiatric disorders, e.g. schizophrenia, it often does not. Historical and contemporary accounts alike describe *self-disturbances* – a blunted presence in the world, the illusion of alien control over actions, derealization, and auditory verbal hallucinations^6,7^. Experimental extensions of the rubber-hand paradigm show that patients from the schizophrenia spectrum are more susceptible to Ownership illusions^8^, suggesting a weakened boundary between *self* and *non-self*. Findings are mixed, however, with some studies attributing the effect to demand characteristics, that is, participants adjusting their responses to what they believe the experimenter expects, or hypnotizability rather than a genuine deficit in multi-sensory integration^9,10^. Hur et al.^11^ suggested that the altered SA in schizophrenia implies an enhanced self-awareness rather than a diminished sense of self, whereas Klaver & Dijkerman^12^ suggested that schizophrenic patients exhibit “weaker stored body representations” and they rely more on external stimuli to make inference about their perceptual experience. A subtler version of these anomalies also surfaces in people scoring high in the domain of *schizotypy*, a personality trait reflecting latent proneness to psychosis, thereby underlining the idea of a schizophrenia spectrum or continuum of psychosis^13^. Elevated schizotypy predicts mild self-disturbances, unusual perceptual experiences, and, importantly, physiological markers reminiscent of schizophrenia. Studying this non-clinical population on the higher end of spectrum therefore offers a window onto mechanisms that might later shed light on the disorder itself.

A converging explanation for these behavioral and experiential anomalies is that the predictive mechanism that normally labels sensations as self‑generated or external runs with reduced precision along the psychosis continuum. Computationally, this mechanism is formalized by the Internal Forward Model: every motor command is accompanied by an efference copy that forecasts its sensory outcome. When the forecast is imprecise or weighted too strongly, self‑produced events are no longer attenuated and can be mis‑tagged as alien^14^. Clinical work supports this view: in schizophrenia the prospective component of Agency (predictive action binding) is weakened, and patients correspondingly rely more on retrospective cues^15^. This predictive deficit manifests electrophysiologically as reduced speaking-induced suppression, a phenomenon where the brain normally dampens its response to self-generated sounds. Specifically, when healthy individuals speak, their auditory cortex suppresses the N1 component of the auditory event-related potential (an early neural marker of sound processing) in response to their own voice. This N1 suppression reflects the brain’s ability to distinguish self-generated from externally generated auditory input. However, in schizophrenia, this suppression mechanism is impaired: patients show larger N1 amplitudes to their own speech than healthy controls, as though the brain fails to flag self-generated sound as internally caused^16–18^. Comparable, though milder, deficits turn up in first-degree relatives, prodromal and high schizotypy individuals^19,20^. Because predictive coding errors are thought to underlie hallucinations, internally generated speech (inner speech) that feels external, measuring N1 suppression provides a direct neural marker of self-monitoring. Yet most work has used simple Talk–Listen paradigms with unmanipulated feedback (where participants speak and hear their own voice, compared to passive listening), so they probe predictive mechanisms only under conditions of largely accurate, veridical feedback. Building on the Talk-Listen framework, the present study uses the Rubber Voice Illusion as an “active challenge” to the forward model. By replacing veridical feedback with a perfectly synchronous but acoustically stranger voice, the RVI stresses predictive coding more strongly than standard Talk–Listen designs, allowing us to map the link between illusion susceptibility, speaking-induced suppression, and psychosis-proneness, as indexed by two questionnaires: Schizotypal Personality Questionnaire^21^ and Peters et al. Delusions Inventory^22^.

The voice carries two primary acoustic cues: pitch, driven by vocal-fold vibration rate (fundamental frequency or F0), and vocal-tract length, shaping timbre. When feedback voice differs from expectation, speakers unconsciously shift their own F0 to compensate, a behavioral fingerprint of how strongly they treat the heard voice as self-generated^5,23,24^. RVI studies show that if the stranger voice is close in pitch, participants adapt towards it; if it is far off, adaptation diminishes^25,26^. Such pitch shifts therefore provide an implicit, objective readout of SO and SA.

In the present study we fused the classic Talk–Listen paradigm with the Rubber Voice Illusion so that we could track, in a single design, how auditory self‑prediction, perceived voice-ownership and schizotypal personality traits interact. While their brain activity was recorded from 64 scalp electrodes, participants repeatedly produced the syllable /ah/ or /bi/ and heard one of three real‑time feedback conditions: (i) a veridical condition that returned their own unaltered voice; (ii) a stranger‑match condition that delivered a temporally and phonetically congruent stranger voice, expected to elicit the illusion; and (iii) a stranger‑mismatch condition in which the same alien voice was semantically incongruent to their own voice (/bi/), serving as a control that should abolish the illusion. After each block the participants rated their sense of Ownership and Agency over the heard voice, allowing us to capture explicit illusion strength, whereas pre-to-post shifts in fundamental frequency (F0) provided an implicit signature of vocal adaptation. Concurrently, we quantified speaking‑induced suppression of the auditory N1 component, a neural index of forward‑model precision.

We predicted that the synchronous stranger feedback would induce the RVI: (1) participants report positive ratings of Ownership and Agency, and nudge their own F0 toward the stranger’s pitch, whereas the mismatched condition does not. At the neural level, we expected (2) the canonical pattern of N1 suppression for self‑produced speech, but with gradation: strongest suppression for veridical self‑feedback, intermediate for the illusion‑inducing congruent condition, and minimal in the incongruent condition. If the forward model indeed mediates the illusion, individuals who showed larger Ownership ratings and greater F0 shifts should also display larger N1 suppression. Finally, (3) drawing on psychosis‑continuum theory, we anticipated that high‑schizotypy participants would both succumb more readily to the illusion and exhibit reduced N1 suppression, revealing latent self‑monitoring deficits that parallel those observed clinically in schizophrenia and other disorders of the spectrum.

By merging a perceptual illusion with a well-characterized neural marker (N1), the present experiment directly probes how predictive coding maintains the boundary between self and other, and how that mechanism falters along the psychosis continuum. The RVI thus extends the body Ownership toolbox into the auditory domain, providing both subjective and objective representations of the minimal self. Confirming our hypotheses would strengthen the case that impaired sensory suppression is not merely a symptom of chronic clinical illness but a continuous, measurable trait that tracks individual vulnerability. As such, the paradigm offers a concise, scalable tool to study how Illusions of the Mind can illuminate and help us understand neuropsychiatric risk.

## Results

### Behavioral data

Behavioral data includes subjective questionnaires (see Supplementary Fig. S1 online) and objective measures (F0 shift in semitones). Veridical auditory feedback consisted of participants’ own unaltered voices, recorded during the training phase. In the two stranger feedback conditions, we presented pre-recorded actor voices (male: 147 Hz; female: 294 Hz), time-locked to each speech onset. These pitch values were chosen to ensure an upward shift relative to each participant’s natural voice, thereby enhancing the perceptual difference, warranting the effect of RVI. The stranger-match condition delivered phonemically congruent feedback (produced and heard syllables identical), whereas the stranger-mismatch condition delivered phonemically incongruent feedback (produced /bi/, heard /ah/). In both cases the temporal alignment was preserved. Results are reported for both grouping schemes, using Schizotypal Personality Questionnaire^21^ (SPQ) and Peters et al. Delusions Inventory^22^ (PDI) cutoffs (see Methods and Supplementary Table S2 online). Participants scored below and above cutoff are grouped in the “control” and “high-schizotypy” group respectively.

#### Subjective rating: illusion questionnaire

**Fig. 1:**
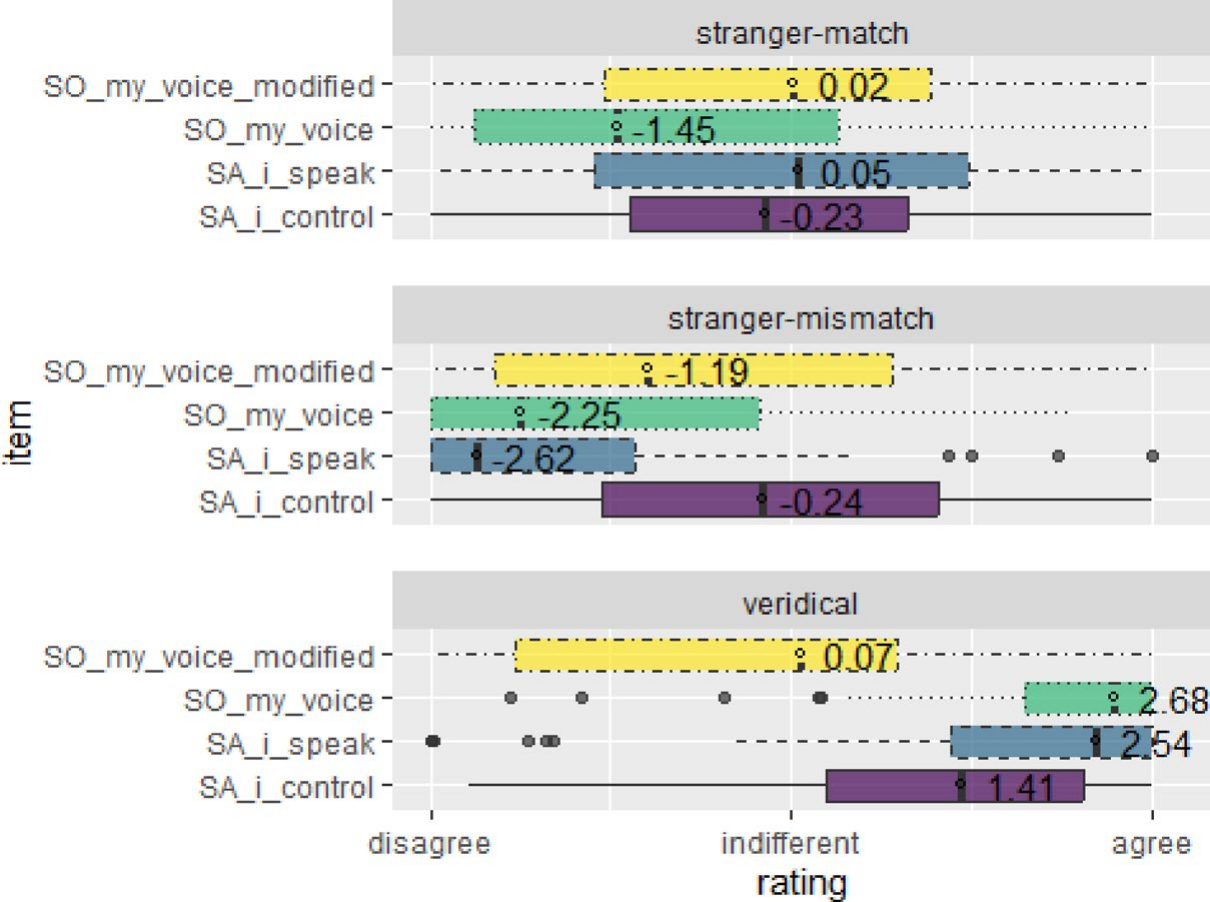
Ownership and agency rating distribution across speaking conditions. Top panel: stranger-match (temporally/phonemically congruent stranger voice); middle: stranger-mismatch (incongruent stranger voice); bottom: veridical (own voice). Four items are shown per panel: SO_my_voice (“It was my voice”), SO_my_voice_modified (“It was a modified version of my voice”), SA_i_speak (“I spoke what I heard”), and SA_i_control (“I controlled the pitch”). Horizontal bars depict interquartile ranges, central markers indicate medians, and points show extreme values. Ratings range from − 3 (disagree) to + 3 (agree), with 0 denoting indifference.

The following analysis of Ownership and Agency ratings collapsed across schizotypy groups. Four items measured voice perception: two Ownership items assessed whether participants felt the voice belonged to them (SO_my_voice: “It was my voice”; SO_my_voice_modified: “It was a modified version of my voice”) and two Agency items assessed perceived Agency (SA_i_speak: “I spoke what I heard”; SA_i_control: “I controlled the pitch”) were highest when participants heard their own unaltered voice, lower for the synchronous stranger voice, and lowest for the asynchronous stranger voice (Fig. 1; Table 1). Repeated measures ANOVA revealed a significant main effect of condition, *F*(1.77, 104.39) = 107.86, *p* < .001, η²G = 0.25. There was also a significant main effect of question, *F*(2.68, 158.34) = 4.09, *p* = 0.010, η²G = 0.02, and a significant condition × question interaction emerged, *F*(4.14, 244.40) = 33.84, *p* < .001, η²G = 0.15. Post-hoc pairwise comparisons using Bonferroni correction revealed that SO_my_voice was highest in the veridical condition (*M* = 2.22) compared with both stranger conditions (match: *M* = − 1.02; mismatch: *M* = − 1.59; both *ps* < 0.001), with the match condition also receiving significantly higher ratings than mismatch (*p* = 0.013). SO_my_voice_modified hovered around neutrality across all conditions (veridical: *M* = − 0.41; match: *M* = − 0.05; mismatch: *M* = − 0.75), with only the comparison between the two stranger conditions reaching significance (*p* = 0.004). SA_i_speak showed the clearest condition effect (veridical: *M* = 1.83; match: *M* = − 0.05; mismatch: *M* = − 1.89), with all pairwise contrasts significant (all *ps* < 0.001). For SA_i_control, veridical ratings were moderately positive (*M* = 1.22) whereas both stranger conditions were near neutral (match: *M* = − 0.23; mismatch: *M* = − 0.20). After Bonferroni correction, veridical ratings significantly exceeded both stranger conditions (both *ps* < 0.001), while the two stranger conditions did not differ from each other (*p* = 1.00).

**Table 1:** The number of participants endorsing the rubber voice illusion (RVI, rating > 0), and descriptive statistics for ownership and agency items across conditions.

Condition	*n* RVI		All blocks		Block 1		Block 2		Order 1		Order 2	
Veridical	*n* Yes	*n* No	Mdn	M (SD)	Mdn	M (SD)	Mdn	M (SD)	Mdn	M (SD)	Mdn	M (SD)
SO_my_voice	57	3	2.68	2.22 (1.17)	2.95	2.17 (1.27)	2.85	2.27 (1.13)				
SO_my_voice_modified	30	30	0.07	− 0.41 (1.85)	0.00	− 0.29 (2.08)	0.00	− 0.53 (1.93)				
SA_i_speak	53	7	2.54	1.83 (1.70)	2.65	1.70 (1.92)	2.67	1.96 (1.62)				
SA_i_control	48	12	1.41	1.22 (1.51)	1.65	1.24 (1.70)	1.46	1.20 (1.73)				
Stranger− match												
SO_my_voice	19	41	− 1.45	− 1.02 (1.81)	− 1.78	− 1.07 (1.92)	− 1.55	− 0.97 (1.96)	− 1.68	− 1.04 (2.05)	− 1.78	− 0.99 (1.82)
SO_my_voice_modified	30	30	0.02	− 0.05 (1.78)	0.43	− 0.11 (1.90)	0.10	0.00 (1.89)	0.56	0.38 (1.81)	− 0.68	− 0.54 (1.87)
SA_i_speak	30	30	0.05	− 0.05 (1.99)	0.53	0.08 (2.21)	− 0.12	− 0.19 (2.11)	0.14	0.02 (2.37)	0.27	− 0.14 (1.91)
SA_i_control	25	35	− 0.23	− 0.23 (1.58)	− 0.01	− 0.20 (1.76)	− 0.03	− 0.25 (1.58)	− 0.23	− 0.23 (1.74)	0.00	− 0.23 (1.59)
Stranger− mismatch												
SO_my_voice	13	47	− 2.25	− 1.59 (1.57)	− 2.34	− 1.38 (1.88)	− 2.69	− 1.81 (1.57)	− 2.08	− 1.28 (1.84)	− 2.82	− 1.87 (1.61)
SO_my_voice_modified	23	37	− 1.19	− 0.75 (1.84)	− 0.93	− 0.65 (1.98)	− 1.03	− 0.84 (1.91)	− 1.02	− 0.72 (2.00)	− 0.98	− 0.77 (1.90)
SA_i_speak	8	52	− 2.62	− 1.89 (1.48)	− 2.77	− 1.77 (1.76)	− 2.83	− 2.01 (1.55)	− 2.46	− 1.40 (1.86)	− 2.98	− 2.32 (1.32)
SA_i_control	26	34	− 0.24	− 0.20 (1.72)	− 0.27	− 0.33 (1.82)	− 0.13	− 0.06 (1.86)	0.29	− 0.04 (1.89)	− 0.42	− 0.33 (1.79)

There is no data for randomization order of the veridical condition since it was always presented last.

Although half the participants gave positive ratings (> 0) to the Agency and Ownership questions in the stranger‑match condition (Table 1), mean scores did not differ from indifference. Ratings of Agency and Ownership tended to be numerically lower in Block 2 than in Block 1 across most conditions (Table 1). However, this apparent decline over time was not statistically significant (*F*(1, 59) = 1.10, *p* = .30, η²G = 0.001). Mixed-design ANOVAs examining SPQ and PDI as between-subjects factors revealed different patterns of moderation (see Supplementary Table S3-S5 online). For SPQ, there were no significant main effects of group and no interactions with the experimental conditions (*p*s > 0.10), suggesting that schizotypy levels did not meaningfully alter participants’ subjective responses to the voice manipulation paradigm. In contrast, the PDI analysis showed no main effect of group but revealed a significant PDI × question interaction (*p* = 0.020), indicating that individuals with high delusion-proneness differed from controls in their response patterns across the four voice perception items.

#### Objective rating: fundamental frequency adaptation

Across the whole sample, exposure to the synchronous stranger voice drove a reliable upward pitch drift from 185.41 Hz (Pre) to 200.25 Hz (Post), in the direction of the higher-pitched stimulus (male: 147 Hz, female: 294 Hz). Controls showed a numerically smaller F0 shift compared to high-schizotypy participants in the stranger match condition (SPQ cutoff: 1.29 vs. 1.42 semitones, PDI: 1.30 vs. 1.41 semitones, see Supplementary Table S6 online). F0 adaptation emerged only when feedback was temporally and phonetically congruent. No pitch shift occurred in the mismatch condition (194.94 → 196.95 Hz). The following analysis only includes the two stranger conditions.

**Fig. 2:**
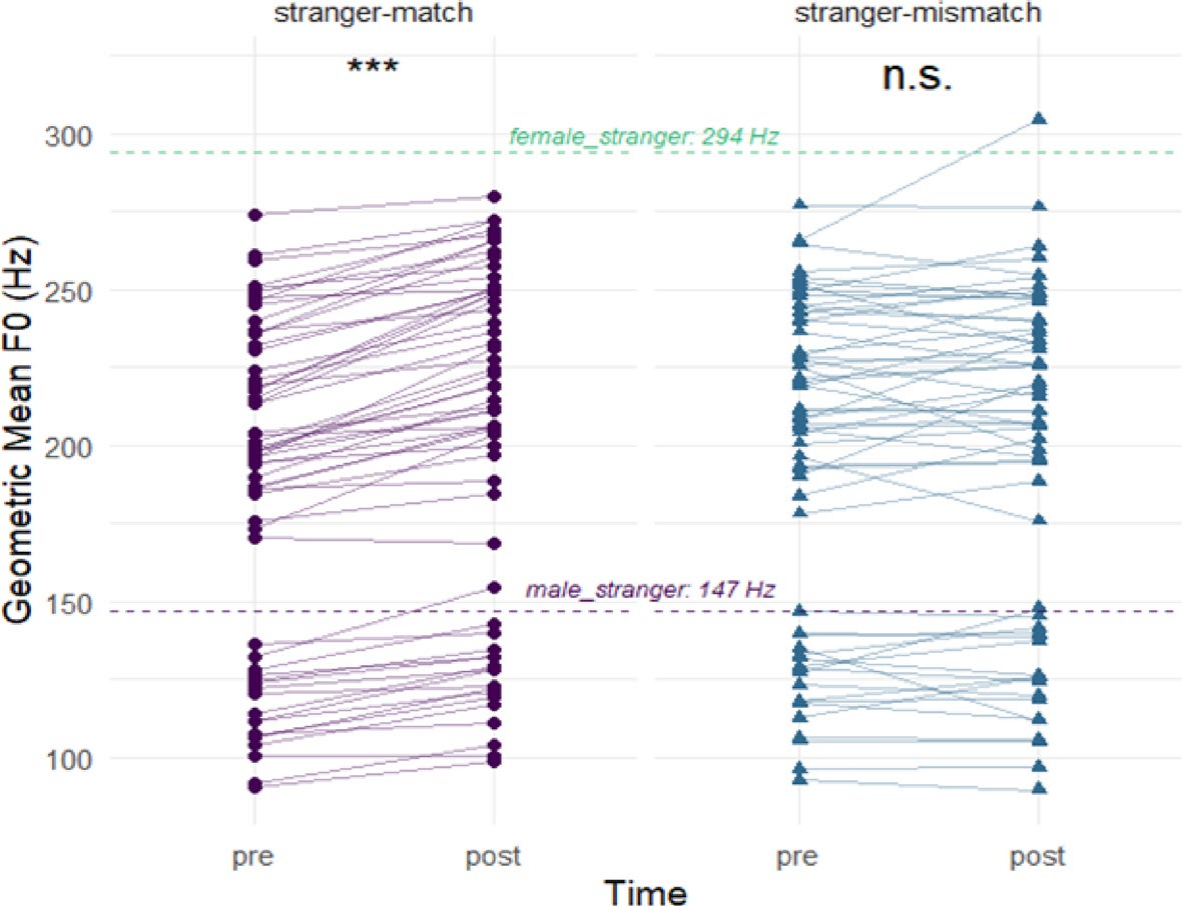
Fundamental-frequency (F0) shifts following exposure to matched versus mismatched stranger voices. Left panel with circle pairs: stranger-match condition; right panel with triangle pairs: stranger-mismatch condition. Each point pair shows an individual’s geometric-mean F0 (Hz) before (pre) and after (post) each condition, connected by a line. Horizontal dashed lines mark the male (147 Hz), and female (294 Hz) stranger voices used in the experiment as feedback.

We ran two repeated-measures ANOVAs with condition (stranger-match vs. stranger-mismatch) as the within-subject factor; group was defined using either the SPQ or PDI cutoff in separate models. Both analyses showed a robust main effect of condition (SPQ cutoff: *F*(1, 58) = 54.40, *p* < .001, η²G = 0.29, PDI cutoff: *F*(1, 58) = 55.44, *p* < .001, η²G = 0.30), with F0 shift larger in match condition (Fig. 2). There was no significant main effect of group (SPQ cutoff: *F*(1, 58) = 0.10, *p* = .755, η²G < 0.01, PDI cutoff: *F*(1, 58) = 0.63, *p* = .430, η²G = 0.006) and no condition × group interaction (SPQ cutoff: *F*(1, 58) = 1.33, *p* = .253, η²G = 0.01, PDI cutoff: *F*(1, 58) = 2.46, *p* = .122, η²G = 0.018).

**Fig. 3:**
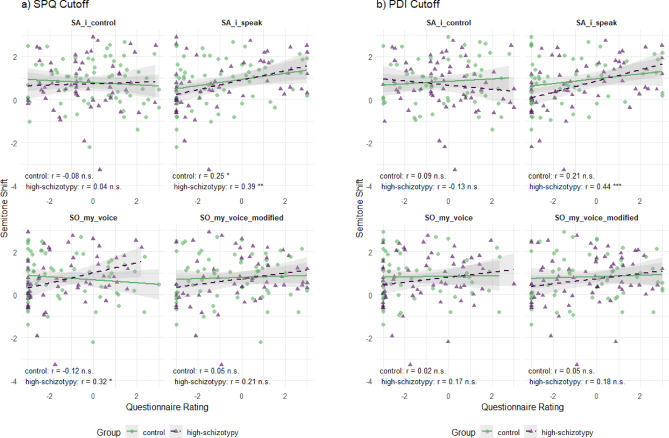
Relationship between pitch shift and subjective agency/ownership ratings by group. (**a**) Groups separated with the SPQ cutoff; (**b**) with the PDI cutoff. Each panel plots F0 shifts in semitone (y-axis) against questionnaire ratings (x-axis) for the four items: SA_i_control, SA_i_speak, SO_my_voice, and SO_my_voice_modified. Green circles + solid lines = controls; purple triangles + dashed lines = high-schizotypy. Lines show group-wise linear fits with 95% confidence bands. Pearson’s r and their significant levels are displayed in each subplot.

We correlated the Agency and Ownership ratings in the behavioral illusion questionnaires with the F0 shift (Fig. 3) to further investigate the effect of induced Ownership and Agency on F0 shift, regardless of the experimental condition (since some participants experienced the RVI even in the mismatch condition). For the analysis with SPQ cutoff, control participants showed a significant positive correlation between speaking Agency ratings (SA_i_speak) and pitch shift (*r* = .25, *p* < .05), indicating that those who felt greater control over their speech production demonstrated larger pitch adjustments. This relationship was even stronger in the high-schizotypy group (*r* = .39, *p* < .01), suggesting that individuals with psychotic-like experiences may be more sensitive to the relationship between perceived Agency and vocal adaptation. Additionally, high-SPQ participants showed a significant correlation between voice-ownership (SO_my_voice) and pitch shift (*r* = .32, *p* < .05), indicating that stronger feelings of voice-ownership were associated with greater vocal accommodation. The PDI analysis revealed the most robust finding, with high-schizotypy participants demonstrating a strong positive correlation between speaking Agency and semitone shift (*r* = .44, *p* < .001).

### Neurophysiological data

#### N1 amplitudes

**Fig. 4:**
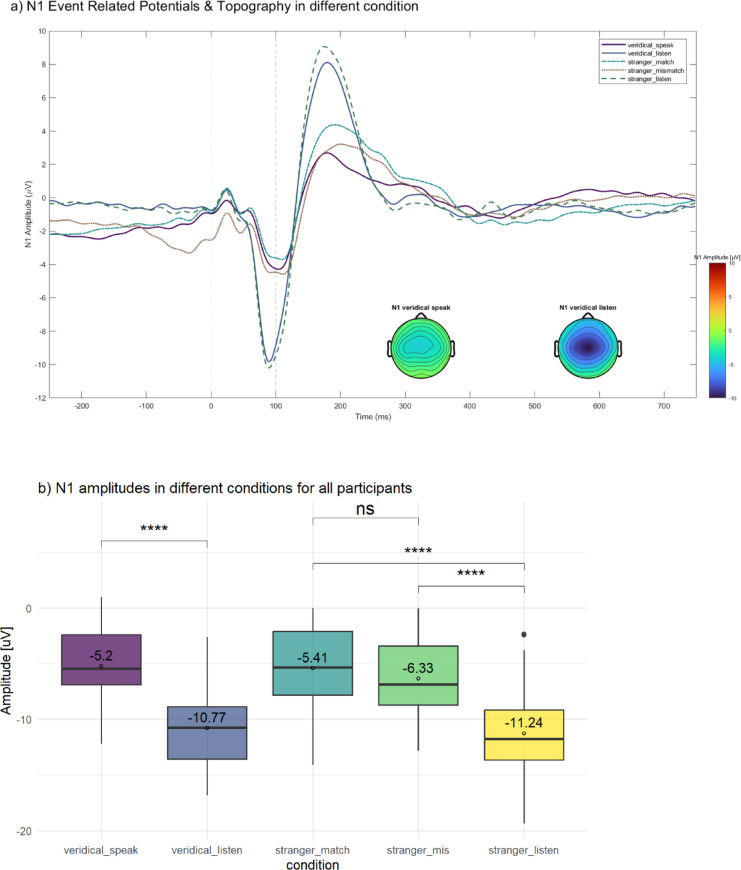
N1 suppression to Talk (self-generated) versus listen across conditions. (**a**) Grand-average ERPs (at Cz) show the N1 component across conditions. Scalp maps depict N1 topography for veridical Talk and Listen, illustrating reduced auditory responses during speaking. (**b**) Boxplots of N1 amplitudes (µV) across conditions. Talk conditions (veridical_speak, stranger_match, stranger_mis) exhibit smaller N1s than their Listen counterparts (veridical_listen, stranger_listen); significance markers indicate pairwise contrasts (**** *p* < .0001; ns = non-significant).

N1 amplitudes were larger during listening than speaking, confirming the effect of speaking-induced suppression (Table 2; Fig. 4). Mean (± SD) values were − 11.24 ± 4.11 µV for stranger-listen and − 10.77 ± 3.52 µV for veridical-listen, compared with − 5.41 ± 3.76 µV (stranger-match speak), − 6.33 ± 3.20 µV (stranger-mismatch speak) and − 5.20 ± 3.28 µV (veridical-speak).

A repeated measures ANOVA examining N1 amplitude across all participants revealed a significant main effect of condition, *F*(4, 236) = 101.92, *p* < .001, η²G = 0.36. Post-hoc pairwise comparisons using Bonferroni correction confirmed that N1 amplitudes were significantly larger (i.e., more negative) during listening compared to speaking. Specifically, veridical-listen differed from veridical-speak (mean difference = 5.56 µV, *p* < .001), and stranger-listen differed from both stranger-match (mean difference = 5.83 µV, *p* < .001) and stranger-mismatch (mean difference = 4.92 µV, *p* < .001). No significant differences were observed between the two listening conditions (e.g., stranger-listen vs. veridical-listen, *p* = .95).

While our primary analyses focus on N1 suppression as the main dependent measure, group comparisons of N1 amplitudes are reported separately in the Supplementary Figures S7–S9 online.

#### N1 suppression

Overall, N1 suppression was strongest in the veridical and stranger-match conditions, and weakest in the stranger-mismatch condition (Table 2). When expressed in µV, mean suppression values for all participants were 5.56 µV (veridical), 5.83 µV (stranger-match), and 4.92 µV (stranger-mismatch). The same pattern is seen in normalized percentage suppression: 52.81% for veridical, 54.62% for stranger-match, and 40.37% for stranger-mismatch.

In terms of group differences, suppression was generally higher in controls compared to high-schizotypy participants, especially when grouped by PDI cutoff (Table 2).

**Table 2:** N1 amplitudes and speaking-induced suppression across conditions and groups.

N1 amplitudes in µV					
Condition	All participants	SPQ cutoff		PDI cutoff	
	(µV)	Control	High schizotypy	Control	High schizotypy
	Mean (SD)	Mean (SD)	Mean (SD)	Mean (SD)	Mean (SD)
Veridical speak	− 5.20 (3.28)	− 5.14 (3.63)	− 5.27 (2.89)	− 4.29 (3.52)	− 6.05 (2.84)
Veridical listen	− 10.77 (3.52)	− 10.20 (4.10)	− 11.41 (2.64)	− 10.52 (4.03)	− 11.00 (3.03)
Strange- match speak	− 5.41 (3.76)	− 5.21 (4.21)	− 5.65 (3.23)	− 4.44 (3.86)	− 6.32 (3.49)
Stranger-mismatch speak	− 6.33 (3.20)	− 6.00 (3.56)	− 6.70 (2.75)	− 5.31 (3.25)	− 7.28 (2.90)
Stranger listen	− 11.24 (4.11)	− 10.69 (4.50)	− 11.87 (3.59)	− 10.82 (4.43)	− 11.64 (3.81)

Speaking-induced suppression / N1 suppression in µV and in %					
	Talk–listen (µV)	Control	High schizotypy	Control	High schizotypy
Veridical	5.56 (3.13)	5.06 (3.12)	6.14 (3.09)	6.22 (3.71)	4.95 (2.36)
Stranger-match	5.83 (3.04)	5.49 (2.86)	6.22 (3.24)	6.38 (3.34)	5.32 (2.69)
Stranger-mismatch	4.92 (4.15)	4.70 (3.57)	5.17 (4.78)	5.50 (3.60)	4.37 (4.60)

	(1-Talk/Listen)*100%	Control	High schizotypy	**control**	High schizotypy
Veridical	52.81 (24.91)	52.06 (26.15)	53.66 (23.86)	60.79 (28.16)	45.34 (18.99)
Stranger-match	54.62 (24.51)	56.22 (25.65)	52.80 (23.48)	62.25 (25.48)	47.49 (21.61)
Stranger-mismatch	40.37 (36.07)	44.53 (29.12)	35.62 (42.72)	51.14 (28.15)	30.30 (40.03)

The table presented reflects N1 suppression across various conditions, categorized by control and high schizotypy groups based on SPQ and PDI cutoffs. N1 suppression is reported in both microvolts (µV) and as a normalized percentage of suppression (1-Talk/Listen)*100.

A 3 × 2 (condition x group: SPQ cutoff) mixed-design ANOVA was conducted on N1 amplitude suppression in percentages. Mauchly’s test indicated that the assumption of sphericity had been violated for the main effect of condition, χ²(2) = 22.07, *p* < .001, therefore degrees of freedom were corrected using Greenhouse-Geisser estimates of sphericity (ε = 0.75). The analysis revealed a significant main effect of condition, *F*(1.50, 87.16) = 6.35, *p* = 0.006, η²*G* = 0.047, indicating that N1 amplitude percentages differed significantly across the three suppression conditions. Post hoc paired t-tests with Bonferroni correction indicated that N1 suppression was significantly greater in the stranger-match than in the stranger-mismatch condition, *t*(59) = 3.15, *p_adj* = 0.008. Veridical and stranger-match conditions did not differ significantly after correction (*p_adj* = 1.00), nor did veridical and mismatch conditions (*p_adj* = 0.06). There was no significant main effect of group, *F*(1, 58) = 0.41, *p* = .526, η²*G* = 0.004, and no condition × group interaction, *F*(1.50, 87.16) = 0.73, *p* = .450, η²*G* = 0.006, indicating that the pattern of N1 suppression across conditions did not differ between groups separated by SPQ cutoff value. A similar analysis using PDI cutoff also revealed a significant main effect of condition, *F*(1.50, 87.23) = 6.31, *p* = 0.006, η²*G* = 0.051. Post hoc comparisons showed the same pattern as in the SPQ analysis: greater suppression in the stranger-match than the stranger-mismatch condition, with no significant differences between the other pairwise comparisons. In contrast to the SPQ analysis, there was a significant main effect of PDI group, *F*(1, 58) = 10.94, *p* = 0.002, η²*G* = 0.088, indicating that participants with high PDI scores exhibited higher overall N1 suppression patterns compared to those with low PDI scores. The condition × PDI group interaction was not significant, *F*(1.50, 87.23) = 0.29, *p* = .685, η²*G* = 0.002.

**Fig. 5:**
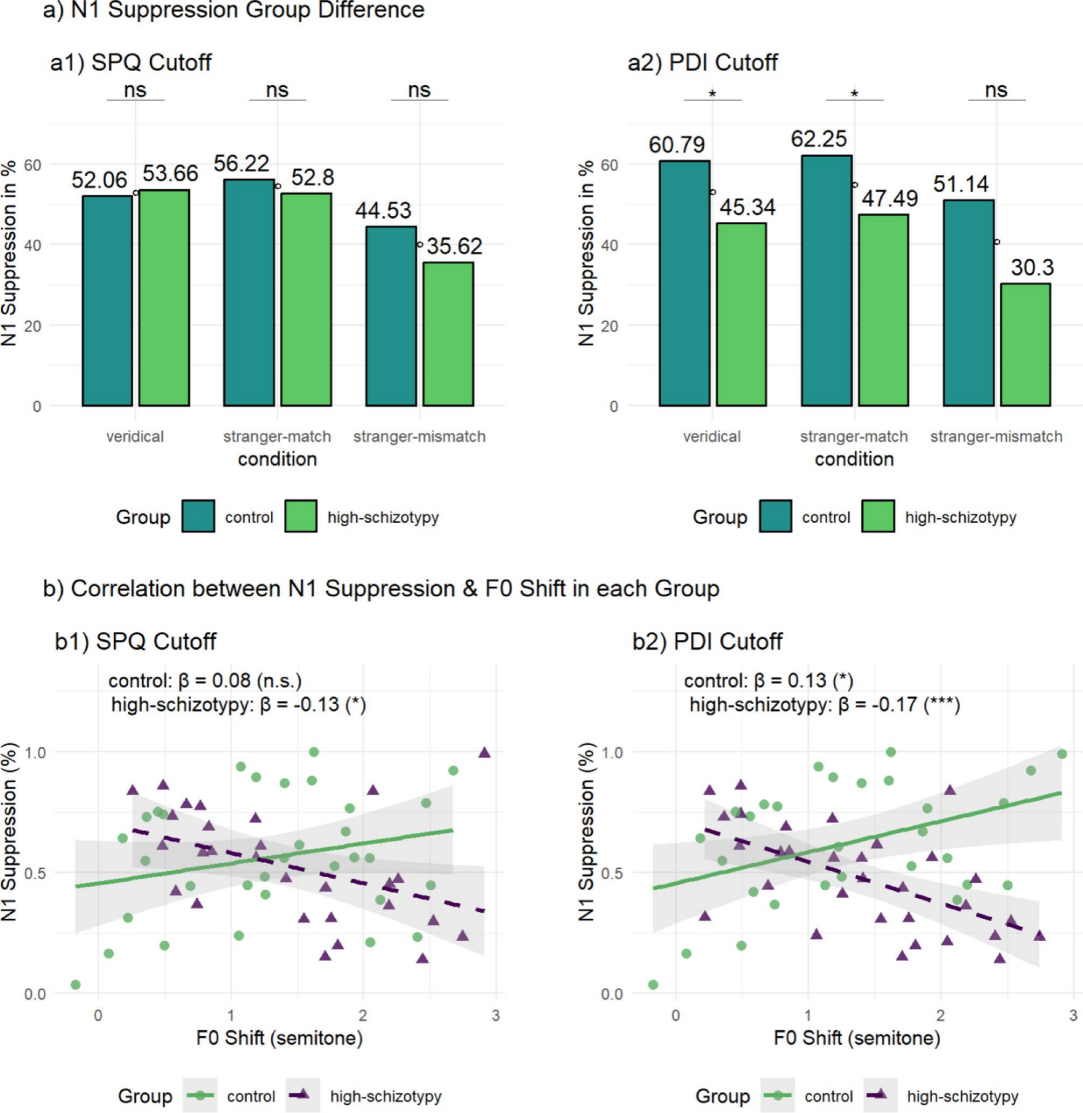
N1 suppression by condition and group, and its relation to pitch shift. (**a1**) SPQ cutoff and (**a2**) PDI cutoff: Percentage N1 suppression ((1 − Speak/Listen) × 100%) for veridical, stranger-match, and stranger-mismatch conditions, shown separately for control and high-schizotypy groups. Asterisks indicate significant group differences (*p < .05; ns = non-significant). (**b1**) SPQ cutoff and (**b2**) PDI cutoff: Scatterplots of N1 suppression (%) versus F0 shift (semitones) within each group, with separate linear fits and 95% confidence bands. Slopes are annotated (β), illustrating the trends in each group. *p*-values were adjusted for multiple comparisons using the Bonferroni correction method.

To further examine group differences in N1 suppression expressed in percentage within each condition, post-hoc pairwise comparisons were conducted using independent-samples Wilcoxon rank-sum tests, with Bonferroni correction applied for multiple comparisons (Fig. 5a). There were significant group differences for veridical suppression (*p* = 0.029) and match suppression (*p* = 0.050), but not for mismatch suppression (*p* = 0.094).

We modeled the F0 shift–N1 suppression relation separately in controls and high-schizotypy participants using simple linear (ordinary-least-squares) regression to test whether its direction and strength differed by group (Fig. 5b). Using the PDI cutoff, for the control group, the regression model was significant, *F*(1, 27) = 5.92, *p* = 0.022, with F0 shift positively predicting N1 suppression (β = 0.13, *t* = 2.43, *p* = 0.022). The model explained approximately 18% of the variance (*R²* = 0.18, adj. *R²* = 0.15). In contrast, for the high-schizotypy group, the model was also significant but showed an opposite effect, *F*(1, 29) = 15.2, *p* < .001. Here, F0 shift negatively predicted N1 suppression (β = − 0.17, *t* = − 3.90, *p* < .001), accounting for about 34% of the variance (*R²* = 0.34, adj. *R²* = 0.32). In a similar analysis using the SPQ cutoff, the model did not reach statistical significance for the control group, *F*(1, 30) = 2.12, *p* = .156. F0 shift positively predicted N1 suppression with a non-significant trend (β = 0.08, *t* = 1.46, *p* = .156), accounting for 7% of the variance (*R²* = 0.07, adj. *R²* = 0.03). For the high-schizotypy group, the model was significant, *F*(1, 26) = 5.49, *p* = 0.027, with F0 shift negatively predicting N1 suppression (β = − 0.13, *t* = − 2.34, *p* = 0.027), and the model explaining 17% of the variance (*R²* = 0.17, adj. *R²* = 0.14).

### Hierarchical models of perception during the rubber voice illusion and its neural correlates

A hierarchical regression (Table 3) approach showed that vocal F0 shift alone (m1/m6) explained little variance in N1 suppression (adj. R² ≈ –0.015). Predictors were entered hierarchically based on their proximity to low-level sensory prediction: (1) vocal F0 shift (implicit motor-acoustic prediction), (2) schizotypy group (trait factor), (3) F0-by-group interaction, and (4) subjective Ownership and Agency ratings (explicit, metacognitive measures). This order allowed us to test the incremental explanatory power of higher-level traits and self-reports.

Adding the group main effect did not improve the SPQ hierarchy (m2 vs. m1: Δ*F*(1, 56) = 0.28, *p* = .596, ΔAIC = + 1.73) but did so for the PDI hierarchy (m7 vs. m6: Δ*F*(1, 57) = 7.50, *p* = 0.008, ΔAIC = − 3.72). Crucially, introducing the F0-shift × Group interaction produced a significant jump in fit for both cut-offs, SPQ: Δ*F*(1, 55) = 6.98, *p* = 0.011, ΔAIC = − 3.3; PDI: Δ*F*(1, 56) = 18.8, *p* < .001, ΔAIC = − 19.0, raising adj. R² to 0.069 and 0.284, respectively. Subsequently adding subjective Ownership (SO) and Agency (SA) ratings, either as main effects (m4/m9) or as interactions with group (m5/m10), failed to yield further improvement (largest Δ*F* = 1.22, *p* ≥ .305; ΔAIC ≥ + 1.35).

**Table 3:** Incremental model comparison for predicting N1 suppression from vocal F0-shift, trait group (SPQ or PDI cut-off), and subjective ownership/agency ratings.

Model	Added terms	ΔDF	F	*p*	adj_R2	AIC	ΔAIC
SPQ cutoff							
m1	F0shift	–	–	–	− 0.015	6.44	3.3
m2	+ SPQ main effect	1	0.28	0.60	− 0.029	8.17	5.03
m3	+ F0 × SPQ interaction	1	6.98	0.011*	0.069	3.14	0
m4	+ SO & SA (additive)	2	0.48	0.62	0.051	6.12	2.98
m5	+ SO × SPQ & SA × SPQ	2	1.61	0.21	0.072	6.51	3.37
PDI cutoff							
m6	F0shift	–	–	–	− 0.015	6.44	19.0
m7	+ PDI main effect	1	7.50	0.0084**	0.061	2.72	15.3
m8	+ F0 × PDI interaction	1	18.8	< 0.001***	0.284	− 12.6	0
m9	+ SO & SA (additive)	2	1.22	0.30	0.289	− 11.3	1.35
m10	+ SO × PDI & SA × PDI	2	0.89	0.42	0.286	− 9.28	3.33

The baseline model is N1 suppression ~ F0shift.

## Discussion

Voice-ownership illusions provide a unique window into how predictive mechanisms support the sense of self in the auditory domain, a process thought to be disrupted in auditory hallucinations. In this study, we investigated whether such disruptions are also evident in psychosis-prone individuals, as indexed by Schizotypal Personality Questionnaire (SPQ) and Peters et al. Delusions Inventory (PDI) scores. To test this, we employed the Rubber Voice Illusion, in which participants spoke while hearing either their own voice, a temporally and phonetically matched stranger voice, or a mismatched stranger voice. Subjective experience was measured using ratings of Agency and Ownership, while implicit vocal adaptation was assessed through changes in fundamental frequency (F0) toward the stranger voice. At the neural level, the reduction of auditory N1 amplitudes during Talk conditions (relative to Listen) was our index of auditory-cortical suppression of one’s own speech (speaking-induced suppression). Behaviorally, half the sample endorsed at least some sense of Agency or Ownership when the stranger voice was perfectly matched, in line with the finding from Zheng and colleagues^5^, but ratings hovered near neutrality at the group level, indicating that (a) a brief exposure produces a fragile illusion easily eroded by fatigue, repetition, and presentation order or (b) the self-report perception through subjective questionnaire is inadequate to capture the illusion. In contrast, the vocal-adaptation measure was the more reliable index: participants shifted their F0 toward the stranger’s higher pitch, but only when timing and phonemes matched, suggesting an implicit adoption of the other voice^5,27^ and showing that pitch drift can expose such adoption even when explicit reports are ambiguous. The correlation analyses revealed that subjective ratings of Agency consistently predicted vocal responses. This suggests that F0 shift serves as a reliable implicit measure of Agency and Ownership, as participants who consciously reported greater control over their speech production also demonstrated larger unconscious vocal adaptations to the manipulated feedback. The particularly robust correlations in high-schizotypy groups indicate that F0 shift may be especially prone to the illusion, providing an objective behavioral marker that complements subjective self-report measures. However, hierarchical regressions showed that when F0 shifts, schizotypy group, and their interaction were entered simultaneously, subjective Ownership and Agency ratings offered no incremental explanatory power, whether entered on their own or combined with group. In contrast, letting pitch shift interact with group markedly boosted explanatory power. Thus, early auditory suppression appears to be tuned to an implicit motor-acoustic prediction (how far the speaker’s pitch follows the heard voice) rather than to the explicit, post-hoc introspection that the voice “felt” like one’s own. In other words, the neural and behavioral signatures of the illusion were best captured by the implicit vocal metric in combination with trait proneness, not by conscious retrospective subjective self-reports.

Speaking-induced suppression of the auditory N1 replicated canonical findings. Listening responses were roughly twice as large as speaking responses, confirming the engagement of forward-model mechanisms during self-generated speech. Yet, contrary to our expectation of a graded congruency effect, N1 suppression was similarly strong in the veridical and stranger-match conditions and only modestly reduced in the stranger-mismatch condition, such that differences among the three feedback types were relatively small. This absence of a strong congruency effect suggests that forward-model prediction may operate at a coarse, categorical level, distinguishing “self-generated” from “externally generated” input, rather than encoding fine-grained acoustic or phonemic details. Supporting this, previous work has shown that even sounds triggered by a button press can elicit N1 suppression^28,29^, indicating that the motor-to-sensory prediction system generalizes across different types of self-initiated actions, not only overt speech. Such findings point to a pre-linguistic, sensorimotor mechanism that is sensitive to action–perception contingencies more than to speech content per se. Secondly, the temporal properties of the N1 may inherently limit its sensitivity to phoneme-level mismatch. The N1 component reflects early sensory prediction errors, typically peaking around 100 milliseconds after sound onset, far earlier than the acoustic unfolding of phonemic content. Therefore, even when voice feedback is mismatched at the phonetic level, the auditory cortex may not register this conflict within the narrow window captured by N1, particularly if rapid repetition was present. Together, these findings suggest that N1 suppression reflects a low-level, temporally coarse mechanism that flags the presence of self-initiation, regardless of the specific acoustic fidelity of the feedback.

Notably, the magnitude of suppression uncovered subtle group differences: individuals with high level of delusion‑prone traits displayed reliably smaller suppression, echoing clinical observations in schizophrenia^16,30–32^ and implying that an impaired predictive mechanism is already present in the non‑clinical population^20^. Interestingly, the significant differences were only present using the PDI index, and not the SPQ (even though it shows a similar pattern), possibly highlighting the type of proneness in the non-clinical population to be delusional thinking patterns, and not (yet) perceptual peculiarities. As discussed recently by Mourgues-Codern et al.^33^, the route people take to descend to psychoticism is very individualistic and delusional proneness seems to precede unusual perceptual experience. This is consistent with our finding on why only PDI seems to predict pitch shift and neural suppression in this sub-clinical group.

When the feedback voice is temporally and phonemically congruent, greater N1 suppression correlates with larger upward shifts in vocal pitch toward the stranger’s voice. Interestingly, neither Ownership nor Agency significantly contributed to explaining N1 suppression, as evidenced by the hierarchical regression models, suggesting that these conscious, subjective ratings may be too distal from the underlying neurophysiological mechanisms of sensory prediction. SO/SA questionnaires tap a retrospective, metacognitive reading of the chain of events, whereas both F0 adaptation and N1 suppression hinge on an online, pre-reflective comparator that operates beneath conscious access. As predictive-coding accounts propose, it is this fast efference-copy mechanism that falters along the psychosis continuum; introspective judgments may come later and be noisier, explaining their limited predictive value here. The group-based findings reveal an interesting divergence: in healthy controls, greater F0 shift was associated with greater N1 suppression, suggesting a coherent self-monitoring system where implicit vocal imitation is accompanied by appropriate neural suppression. However, in individuals with high schizotypy, this relationship reversed, such that greater F0 shifts were accompanied by reduced N1 suppression. This pattern implies that while high-schizotypy individuals still exhibit flexible vocal adaptation, readily shifting their pitch toward the stranger’s, they may do so without the corresponding neural suppressions. Ownership illusion studies show that schizophrenia and high‑schizotypy populations overweight external cues when deciding what belongs to the self^8,12^. Our data extend that bias into the auditory‑motor domain: high‑schizotypy speakers implicitly align their pitch with an alien voice even while their forward model fails to flag and attenuate the sounds. This dissociation aligns with theoretical accounts of self-disturbance in schizotypy: their expanded or unstable self-representations may allow them to unconsciously adopt external cues (e.g., pitch) more readily, but with weaker corollary discharge mechanisms, leading to diminished sensory suppression. In short, while the implicit behavior evidence (pitch shift) is present, the monitoring system (N1 suppression) does not reflect it, highlighting a disconnect between action and self-perception. This diminished self-monitoring may illuminate the origins of hallucinations, where self-generated actions and thoughts are not properly recognized as one’s own and suppressed accordingly, and are instead attributed to external sources.

Several methodological factors likely limited illusion prevalence. Ratings decayed from the first to the second block, indicating that novelty or sustained attention is essential for maintaining the altered self‑attribution. Presentation order mattered: exposure to the mismatched voice first dampened subsequent illusion, whereas the reverse sequence primed higher Ownership. The simultaneous display of questionnaire items also encouraged response revision. These findings highlight the limitations of subjective reports and underscore the need for more robust, implicit measures such as F0 shifts. Acoustic idiosyncrasies beyond timing, especially pitch distance and timbre, almost certainly contributed to individual variability; personalizing the stranger voice to lie within each participant’s just‑noticeable pitch difference and spectrally morphing timbre would sharpen multisensory congruence. In the stranger-mismatch condition, participants produced /bi/ while hearing /ah/, so the conditions differed not only in phonetic congruence but also in articulatory configuration. Because these articulation patterns were constant within each condition, they are unlikely to account for the condition-by-group effects on N1 suppression. Nonetheless, this confound represents a limitation of the design. Future work could manipulate phonetic congruence while holding articulatory demands constant. Bone‑conduction cues, omitted in the current study, may have preserved a residual proprioceptive anchor to the true voice, diluting the illusion. Within these constraints, the implicit vocal drift and N1 suppression provide converging evidence that the Rubber Voice Illusion paradigm stress‑tests the auditory forward model more strongly than conventional Talk–Listen contrasts and exposes trait‑linked vulnerabilities. Longitudinal studies could also test whether reduced suppression and heightened implicit vocal adoption predict transition to psychosis. Technically, integrating real‑time voice‑morphing algorithms that adjust pitch and timbre on the fly, alongside bone‑conduction control and prolonged exposure, should deliver a more potent and sustained illusion suitable for patient cohorts.

In summary, exposure to a congruent stranger’s voice can temporarily alter our sense of vocal self-ownership, which is detected by unconscious vocal adaptation and early brain responses. These findings refine our original prediction: while we anticipated a three-way link among F0 shift, Ownership/Agency ratings, and N1 suppression, the hierarchical models reveal a two-tier architecture, implicit motor-acoustic prediction (F0 shift) interfaces directly with early sensory suppression (N1 suppression), and this relationship is reversed in delusion-prone individuals. The Rubber Voice Illusion therefore offers a concise, multi‑modal tool for probing predictive coding of the self-generated actions across the psychosis continuum and furnishes a foundation for mechanistic work on the neural correlates of auditory hallucinations.

## Methods: summary, full details in supplementary

### Participants and power

G*Power 3.1^34^ indicated that 56 participants were required to detect a medium effect (*f* = 0.25) in a 2 (group) × 5 (condition) mixed design with α = 0.05 and 1 – β = 0.80. Sixty-four volunteers (42 women; age = 24.8 ± 4.1 years, range = 19–36) met al.l criteria and completed the study. Recruitment was conducted via university portals, e-mail flyers, and adverts targeting programs in art, theatre, and music, where schizotypal traits are over-represented. An online screening survey delivered the German version of the Schizotypal Personality Questionnaire^35^ (SPQ) and Peters et al. Delusions Inventory^36^ (PDI). Participants scoring ≤ 21.6 on the SPQ (German population mean^35^ formed the control group; those scoring > 21.6 comprised the high-schizotypy group. In a parallel analysis, we also used PDI mean as the cutoff (*M* = 5), since there was no recorded PDI mean value for the German population. All were right-handed native German speakers with normal hearing (confirmed with onsite audiometry test), no history of psychosis, neurological illness, or tinnitus. Four datasets were discarded because of task non-compliance (*n* = 2), earphone malfunction (*n* = 1) or excessive cough artifacts (*n* = 1). Each participant received monetary compensation and gave written informed consent. The experiment was approved by the Ethics committee of the Institute of Psychology of Humboldt-Universität zu Berlin, Germany. All research methods were performed in accordance with relevant guidelines and regulations, including the Declaration of Helsinki. All identifying information has been removed to protect participant privacy.

### Auditory stimuli and real-time feedback system

Using the Praat software^37^, we synthesized the stimulus voices of the actors. Stimuli were /a/ and /bi/ syllables (for match and mismatch condition, respectively). The male stimulus is D3 (147 Hz), and the female stimulus is D4 (294 Hz). The frequency of the stimulus range was adopted from a pilot study. We also recorded and cleaned the participant’s own voice during the training session of the experiment and used it as stimulus in the Listen condition. The participants received either the female or male voice as stimulus, depending on their reported biological sex, and their own voice in separate experimental conditions. The phonetic content, onset and offset of the auditory feedback were congruent with the participants’ own vocalization. Speech was captured with a Shure SM35 headset microphone, pre-amplified by a Great River ME-1NV, and routed to a Tucker-Davis Technologies RP2.1 real-time processor running custom RPvdsEx / MATLAB code. The RP2.1 relayed either the participant’s live voice or pre-recorded male/female actor tokens to a Violectric HPA-V200 headphone amp and E-A-RTONE 3 A insert earphones, whose passive isolation reduced ambient noise and bone-conducted self-voice. Vocal feedback latency was ≈ 4 ms (RP2.1 spec). The system gated recordings on when sound-pressure level exceeded a pilot-derived RMS threshold and off when it fell below the offset threshold, while simultaneously sending TTL triggers to the EEG amplifier to mark trial and vocal onsets, creating a fully closed-loop, real-time audio-EEG acquisition setup.

### EEG acquisition

EEG was recorded with a 64-channel BrainVision system (Brain Products, Germany) at 1 kHz (0.5 µV resolution, ± 16.384 mV range, DC–250 Hz hardware band-pass). Ag/AgCl electrodes in an EasyCap were referenced online to FCz and grounded at AFz; impedances were kept below 20 kΩ using Abralyt gel. Bipolar HEOG/VEOG and peri-oral EMG channels were acquired through a BrainAmp ExG. TTL pulses from the TDT processor tagged stimulus and voice onsets. Experimental scripts and task control were delivered with MATLAB R2022b^38^ and Psychtoolbox^39^.

### Experimental design

The experiment used a mixed design comprised five within-subject conditions (1) veridical Speak: participants say /ah/ and hear their own voice looped back, (2) veridical Listen: passively listen to recordings of their own voice, (3) stranger-match Speak: participants say /ah/ and hear a stranger voice saying /ah/, (4) stranger-mismatch Speak: say /bi/ but hear a stranger voice saying /ah/, and (5) stranger Listen: passively listen to stranger voice saying /ah/. Each condition comprises two 50-trial blocks (4 s/trial), separated by a brief rest, for a total run time of around 1.5 h (3 h including setup). Condition order was pseudo-counterbalanced: stranger-match and stranger-mismatch were alternated, whereas both veridical conditions (Talk and Listen) were always presented last to avoid illusion wash-out (presenting veridical feedback first led to steeper, earlier declines in Agency and Ownership during subsequent stranger-voice blocks). This choice was guided by pilot observations as well as a similar voice-ownership paradigm^5^ in which the order of congruent vs. incongruent feedback modulates illusion strength. In each trial, a fixation “+” appeared for 1 s; participants vocalized after it disappeared, aiming for around 250 ms duration with minimal jaw-lip motion. Talk conditions contained three phases: Pre-no-feedback (10 trials), Feedback (100 trials), Post-no-feedback (10 trials). In Listen conditions auditory stimuli were presented without vocalization. After every Talk block (50 trials), participants rated Agency and Ownership of the heard voice in a questionnaire and were offered a short break. TTL triggers marked stimulus and voice onsets in the EEG.

### Illusion questionnaire

After each 50-trial block, participants rated Ownership (“the voice sounded like mine/like a modified version of mine”) and Agency (“I was producing what I heard/I could control the pitch”) on a − 3 to + 3 visual-analogue scale; three control items assessed localization and source-control illusions. The full questionnaire can be found in Supplementary Figure S1 online.

### Fundamental frequency (Vocal F0)

Praat^37^ extracted F0 mean, converted to logarithmic scale, and averaged geometrically Pre and Post per condition. Vocal adaptation was indexed as semitones change from pre to post (semitone_shift = 12 * log2(post / pre)).

### EEG pre-processing and ERP measurement

EEG preprocessing was performed in MATLAB R2022b^38^ with EEGLAB 2023^40^, the ICLabel add-on, and the CleanRawData (ASR) plugin. Continuous data were first low-pass filtered at 40 Hz with a linear-phase FIR filter, down-sampled to 250 Hz to accelerate ICA, and high-pass filtered at 1 Hz to remove slow drift. Silent pauses were excised and remaining high-variance segments discarded with a ± 3 SD joint-probability test. An extended Infomax ICA was then computed, and the resulting weights were reapplied to the raw recording to preserve full spectral content. Components classified by ICLabel as ocular or muscle with ≥ 0.80 probability, together with any additional speech- or channel-related components identified by visual inspection, were removed. Residual bursts and noisy channels were cleaned with ASR (flatline > 5 s, bad channel criterion = 0.80, burst cut-off = 40), and rejected channels were spherically interpolated. The signal was finally band-passed 1–30 Hz, re-referenced to the common average, and resampled to 500 Hz. Cleaned data were epoched from − 250 to + 750 ms around each event, baseline-corrected with the 250 ms preceding the fixation cross to avoid Readiness Potentials in the Talk conditions (and with the 250 ms baseline preceding the stimulus onset in the Listen conditions). Any trials exceeding ± 80 µV or 3 SD joint-probability were excluded. EOG and EMG channels were removed. The resulting artifact-free epochs constituted the input for ERP analyses. N1 was calculated as the most negative peak 50–150 ms at Cz. Suppression indices were computed as (1-Talk/Listen)*100 for normalized percentage, and as (Talk-Listen) for absolute value. We adopted the percentage-normalized N1 index because it scales suppression to each participant’s Listen baseline, reducing between-subject variance and matching previous research^41,42^.

### Statistical analysis

All statistical analyses were performed in R^43^. Agency/Ownership ratings were analyzed with one-way and two-way repeated-measures ANOVA and post-hoc tests using Bonferroni correction; group effects were examined with mixed ANOVAs. F0 ratios and N1 amplitudes were evaluated with paired sample t-tests and mixed ANOVAs. Correlations used Pearson’s r. Multiple-comparison p-values were Bonferroni-corrected. We ran hierarchical linear regressions in which N1 suppression was first predicted by F0 shift, schizotypy group (PDI or SPQ cut-off) and their interaction, then tested whether adding Ownership and Agency ratings improved the model.

## Supplementary Information

Below is the link to the electronic supplementary material.

Supplementary Material 1

## Data Availability

The datasets generated and analyzed during the current study are available in the RVI repository on GitHub: https://github.com/suongwelp/RVI/.
