# Supplementary material for: Rubber voice illusion exposed neural correlates of voice perception and vocal adaptation across the continuum of psychosis
Source: Sci Rep. 2026 Jan 8;16:1295. doi: 10.1038/s41598-025-34714-9 (PMC12791131; doi:10.1038/s41598-025-34714-9)

Rubber Voice Illusion exposed neural correlates of voice perception and vocal adaptation across the Continuum of Psychosis

Suong Welp[1,2*], Andrea Hildelbrandt[1,3], David A. Magezi[4], Martin Voss[5], and Laura Kaltwasser[6]

[1]Department of Psychology, Carl von Ossietzky Universität Oldenburg, Oldenburg, 26129, Germany
[2]Max Planck Institute for Human Cognitive and Brain Sciences, Leipzig, 04103, Germany
[3]Cluster of Excellence Hearing4all, Carl von Ossietzky Universität Oldenburg, Oldenburg, Germany
[4]Biological Psychology and Neuropsychology, University of Hamburg, Hamburg, 20146, Germany
[5]Department of Psychiatry and Psychotherapy (Charité Campus Mitte), Charité University Medicine and St. Hedwig Hospital, Berlin, 10115, Germany
[6]Humboldt-Universität zu Berlin, Berlin School of Mind and Brain, Berlin, 10117, Germany
*welp@cbs.mpg.de
+these authors contributed equally to this work

Supplementary

Supplementary Figure S1. Voice Perception Questionnaire (German version, translation below)
Participants rated their agreement with seven statements about the voice they heard through headphones, using a visual analog scale ranging from "gar nicht" (not at all, coded as −3) to "vollkommen" (completely, coded as +3), with "weder noch" (neither nor, coded as 0) as the midpoint. The questions indexed perceived Agency (e.g., control over pitch or source), Ownership (e.g., whether the voice felt like their own or a modified version), and localization of the voice.

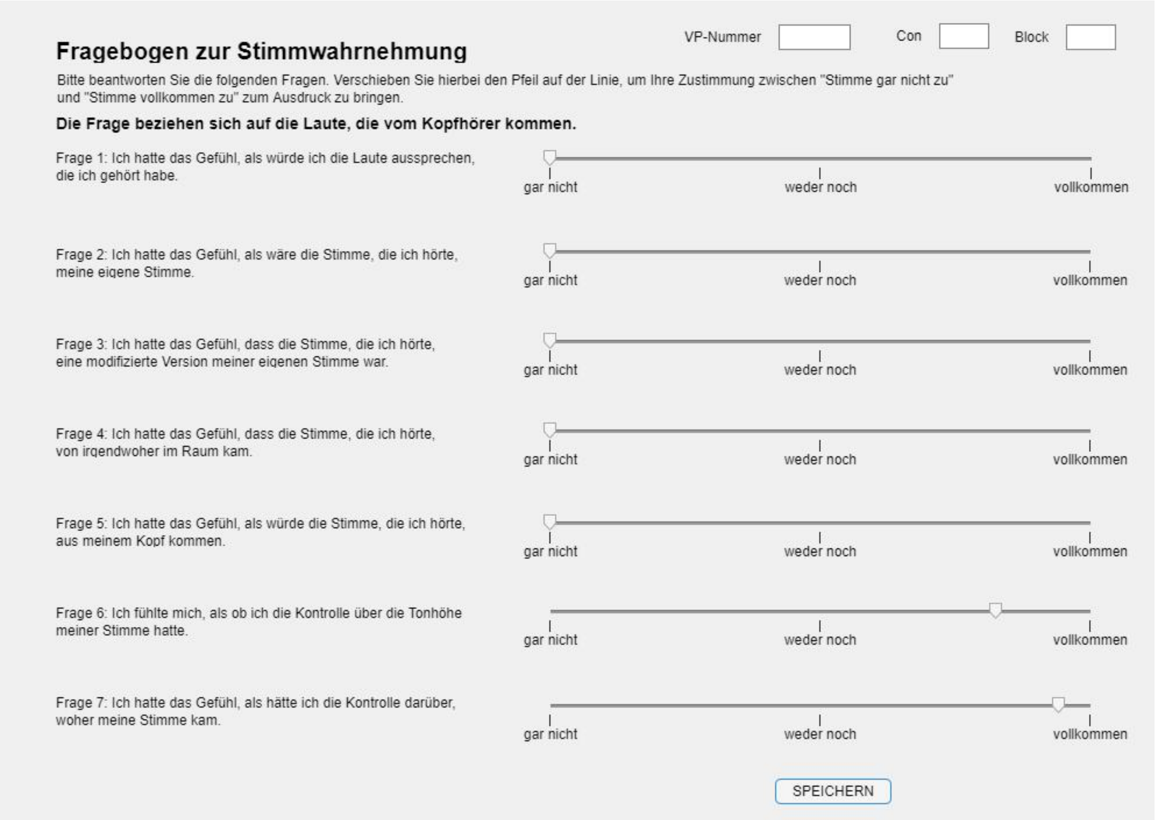

Translation

Please answer the following questions. Move the arrow along the line to express your agreement from "not at all" to "completely."

The questions refer to the sounds coming from the headphones.

Question 1 I had the feeling that I was speaking the sounds that I heard.

Question 2 I had the feeling that the voice I heard was my own voice.

Question 3 I had the feeling that the voice I heard was a modified version of my own voice.

Question 4 I had the feeling that the voice I heard was coming from somewhere in the room.

Question 5 I had the feeling that the voice I heard was coming from inside my own head.

Question 6 I felt as if I had control over the pitch of my voice.

Question 7 I had the feeling that I had control over where my voice was coming from.

Supplementary Table S2. Participant demographics and questionnaire scores by schizotypy group (SPQ and PDI cutoffs).

Displayed are the means, standard deviations (in brackets), and medians for age, gender distribution, and total scores on the Schizotypal Personality Questionnaire (SPQ) and Peters et al. Delusions Inventory (PDI), separated by different grouping (SPQ cutoff vs. PDI cutoff). Gender is shown as female/male count.

			**SPQ Cutoff**				**PDI Cutoff**			
	All participants		control		high schizotypy		control		high schizotypy	
**Variable**	*M (SD)*	*Mdn*	*M (SD)*	*Mdn*	*M (SD)*	*Mdn*	*M (SD)*	*Mdn*	*M (SD)*	*Mdn*
**Age**	24.77 (3.61)	24	24.91 (3.46)	24.5	24.61 (3.83)	23.5	24.8 (3.53)	24	24.7 (3.74)	24
**Gender(female/male)**	42/18		23/9		19/9		20/9		22/9	
**PDI score total**	4.98 (3.61)	5	3.38 (2.72)	3	6.82 (3.67)	6.5	2 (1.22)	2	7.77 (2.77)	7
**SPQ score total**	21.88 (12.89)	20	12.47 (6.19)	14	32.64 (9.69)	29.5	14.8 (9.41)	14	28.5 (12.3)	27

Note. M = Mean*, Mdn* = Median*,* SD = Standard Deviation; m = male; f = female; SPQ = Schizotypal Personality Questionnaire; PDI = Peters et al. Delusions Inventory.

We have a binominal distribution of SPQ. Shapiro-Wilk test was performed and confirmed that the distribution of SPQ departed significantly from normality ($W = 0.957$, $p < .05$). The PDI score is also not normally distributed (Shapiro-Wilk test, $W = 0.938$, $p < .01$).

A linear mixed-effects model tested the effect of presentation order with condition and question as within-subject factors on ratings. Using Satterthwaite degrees of freedom, there was a significant main effect of order, $F(1, 406) = 7.25$, $p = .007$, and no significant interactions. Holm-adjusted pairwise contrasts (order 1 vs. 2 within each condition × question) showed two small but significant differences: Agency (SA_i_speak) in the mismatch condition has higher ratings when the mismatch condition was presented before the match condition ($t(295) = 2.07$, $p = .040$) and Ownership (SO_my_voice_modified) has higher ratings when match condition was experienced before mismatch ($t(295) = 2.06$, $p = .041$).

Post-hoc pairwise comparisons provided more nuanced insights into these group differences. For SPQ, while the overall ANOVA suggested minimal moderation, specific comparisons revealed that high-SPQ individuals showed enhanced sensitivity to certain experimental manipulations. Notably, the high-SPQ group demonstrated a significant difference between match and mismatch conditions for the SO_my_voice item ($p = .010$) that was absent in controls ($p = .660$). Similarly, for the SO_my_voice_modified item, only the high-SPQ group showed a significant difference between match and veridical conditions ($p = .037$), while controls showed no such differentiation. The PDI analysis revealed a different pattern of group moderation, particularly for the SO_my_voice_modified item. Control participants maintained sensitivity to the experimental manipulation, showing a significant difference between match and mismatch conditions ($p = .007$), while the high-PDI group showed no such differentiation ($p = .450$). For other voice perception items, both groups generally showed consistent condition effects.

Supplementary Table S3. Median and mean ratings for each illusion questionnaire item by condition and schizotypy group.

Participants rated each item using a scale from −3 (“not at all”) to +3 (“completely”). The table presents the median (*Mdn*), mean (*M*) and standard deviation (*SD*) ratings for each item—Sense of Ownership (SO_my_voice, SO_my_voice_modified) and Sense of Agency (SA_i_speak, SA_i_control)—across three conditions (veridical, stranger-match, stranger-mismatch), separately for control and high-schizotypy groups using both SPQ and PDI cutoffs.

		**SPQ Cutoff**				**PDI Cutoff**			
		**Control**		**High schizotypy**		**Control**		**High schizotypy**	
Condition	Item	*Mdn*	*M (SD)*	*Mdn*	*M (SD)*	*Mdn*	*M (SD)*	*Mdn*	*M (SD)*
Veridical									
	SO_my_voice	2.66	2.32 (0.99)	2.68	2.09 (1.36)	2.53	1.94 (1.37)	2.98	2.47 (0.90)
	SO_my_voice_modified	0.48	-0.03 (1.95)	-1.08	-0.85 (1.65)	0.48	0.19 (1.68)	-1.27	-0.98 (1.84)
	SA_i_speak	2.74	2.05 (1.59)	2.24	1.58 (1.81)	2.50	1.51 (2.10)	2.56	2.12 (1.17)
	SA_i_control	1.86	1.36 (1.66)	1.16	1.05 (1.32)	1.26	0.86 (1.64)	1.66	1.55 (1.32)
Stranger-match									
	SO_my_voice	-1.75	-1.18 (1.85)	-1.05	-0.83 (1.78)	-2.18	-1.40 (1.79)	-0.79	-0.67 (1.78)
	SO_my_voice_modified	-0.01	-0.27 (1.77)	0.24	0.20 (1.79)	0.47	0.18 (1.81)	-0.08	-0.27 (1.75)
	SA_i_speak	-0.61	-0.31 (1.91)	0.94	0.24 (2.08)	-0.97	-0.50 (2.03)	0.86	0.36 (1.89)
	SA_i_control	0.34	-0.02 (1.73)	-0.70	-0.46 (1.38)	-0.23	-0.28 (1.77)	-0.23	-0.17 (1.40)
Stranger-mismatch									
	SO_my_voice	-2.49	-1.54 (1.75)	-2.23	-1.65 (1.36)	-2.73	-1.76 (1.64)	-1.75	-1.44 (1.52)
	SO_my_voice_modified	-1.29	-0.98 (1.79)	-0.24	-0.47 (1.88)	-1.25	-0.79 (1.89)	-0.92	-0.70 (1.82)
	SA_i_speak	-2.66	-1.89 (1.45)	-2.49	-1.90 (1.53)	-2.66	-2.01 (1.54)	-2.28	-1.78 (1.43)
	SA_i_control	-0.11	0.09 (1.77)	-0.57	-0.52 (1.63)	-0.60	-0.28 (1.75)	-0.18	-0.12 (1.72)

Supplementary Table S4. Mixed-Design ANOVA Results with SPQ and PDI as Between-Subjects Factors

The table reports F-values, degrees of freedom (Greenhouse–Geisser corrected where indicated), p-values, and generalized eta squared (η^2G) for each main effect and interaction in a mixed-design ANOVA. The within-subjects factors were Condition (veridical, stranger-match, stranger-mismatch) and Question (SO_my_voice, SO_my_voice_modified, SA_i_speak, SA_i_control). Between-subjects groupings were based on either SPQ or PDI cutoff scores.

**Effect**	**SPQ Analysis**				**PDI Analysis**			
	F	df	*p*	η^2G	*F*	df	*p*	η^2G
**Main Effects**								
**Group**	0.21	1, 58	0.651	< .01	1.26	1, 58	0.266	0.005
**Condition**	110.38	1.79, 103.85[a]	< .001	0.25	106.24	1.77, 102.71[a]	< .001	0.26
**Question**	4.07	2.69, 156.02[a]	0.01	0.02	4.26	2.68, 155.54[a]	0.008	0.02
**Interactions**								
**Group × Condition**	2.38	1.79, 103.85[a]	0.103	0.01	0.11	1.77, 102.71[a]	0.871	< .01
**Group × Question**	0.78	2.69, 156.02[a]	0.496	< .01	3.54	2.68, 155.54[a]	0.02	0.02
**Condition × Question**	34.05	4.21, 244.18[a]	< .001	0.15	34.3	4.23, 245.05[a]	< .001	0.15
**Group × Condition × Question**	1.36	4.21, 244.18[a]	0.245	0.01	1.81	4.23, 245.05[a]	0.123	0.009

Note. [a]Greenhouse-Geisser corrected degrees of freedom due to sphericity violations.

Supplementary Table S5. Pairwise comparisons for illusion questionnaire items by condition and group (Bonferroni-corrected p-values).

Question	SPQ Group	Match vs Mismatch	Match vs Veridical	Mismatch vs Veridical	PDI Group	Match vs Mismatch	Match vs Veridical	Mismatch vs Veridical
**SA_i_control**	Control	1	0.003	0.003	Control	1	0.026	0.01
	High-schizotypy	1	< .001	0.002	High-schizotypy	1	< .001	< .001
**SA_i_speak**	Control	< .001	< .001	< .001	Control	0.003	< .001	< .001
	High-schizotypy	< .001	0.009	< .001	High-schizotypy	< .001	< .001	< .001
**SO_my_voice**	Control	0.66	< .001	< .001	Control	0.2	< .001	< .001
	High-schizotypy	0.01	< .001	< .001	High-schizotypy	0.079	< .001	< .001
**SO_my_voice_modified**	Control	0.077	1	0.127	Control	0.007	1	0.119
	High-schizotypy	0.075	0.037	1	High-schizotypy	0.45	0.29	1

Note. Significant differences ($p < .05$) indicate condition effects within each group.

Supplementary Table S6. Mean F0 (Hz) pre and post condition, and F0 semitone shift by condition and schizotypy group.

For each stranger condition (match and mismatch), geometric mean F0 (in Hz) and standard deviations before and after each condition, as well as the semitone shift (positive values indicate shift toward the stranger voice) were reported. Results are shown for all participants and split by schizotypy group using both SPQ and PDI cutoffs.

Condition	Group	Pre in Hz (SD)	Post in Hz (SD)	Semitone-Shift (SD)
**SPQ Cutoff**				
**Match**	All participants	185.41 (52.46)	200.25 (55.77)	1.35 (0.78)
	control	184.24 (47.55)	198.33 (50.16)	1.29 (0.80)
	high-schizotypy	186.74 (58.42)	202.44 (62.44)	1.42 (0.78)
**Mismatch**	All participants	194.94 (52.73)	196.95 (54.23)	0.15 (1.08)
	control	194.22 (49.19)	196.64 (47.66)	0.27 (1.06)
	high-schizotypy	195.75 (57.41)	197.31 (61.79)	0.02 (1.11)
**PDI Cutoff**				
**Match**	control	185.13 (51.89)	199.36 (54.62)	1.30 (0.84)
	high-schizotypy	185.66 (53.84)	201.08 (57.73)	1.41 (0.74)
**Mismatch**	control	195.06 (54.44)	198.45 (52.82)	0.36 (1.08)
	high-schizotypy	194.82 (51.97)	195.55 (56.35)	-0.04 (1.07)

Supplementary Figure S7. N1 amplitude comparisons between control and high-schizotypy groups across experimental conditions.

(A) SPQ cutoff and (B) PDI cutoff. Boxplots show N1 amplitudes (μV) for each condition (veridical_speak, veridical_listen, stranger_match, stranger_mis, stranger_listen) separately by group. Horizontal lines represent means, with p-values from independent-samples t-tests shown above each pair (both uncorrected and Bonferroni-adjusted).

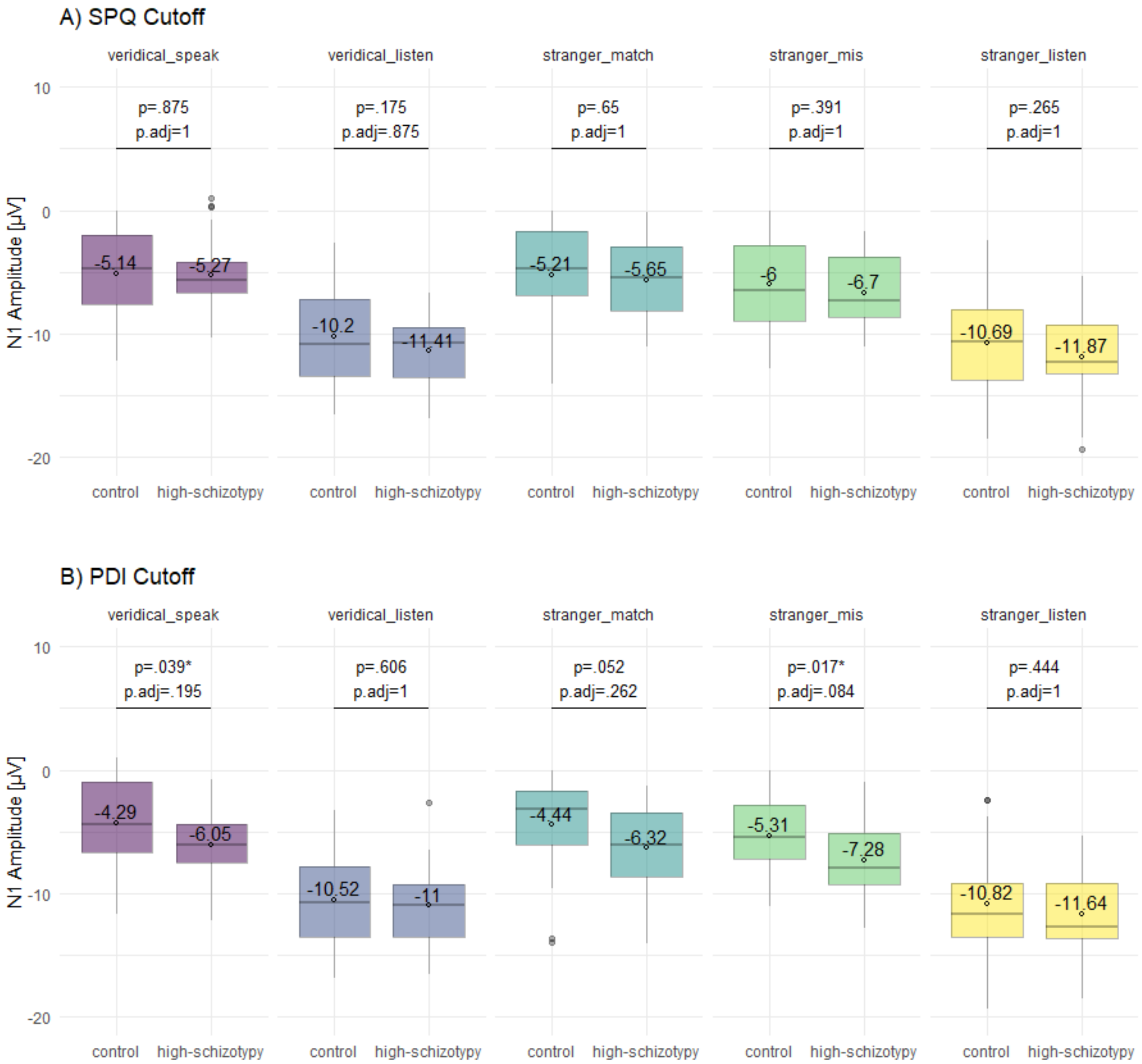

Note. Pairwise t-tests corrected with Bonferroni adjustment for multiple comparisons.

In the PDI-based grouping, high-schizotypy participants consistently showed more negative N1 amplitudes than controls across all speaking conditions, with the largest difference observed in the stranger-mismatch speak condition (−7.28 μV vs. −5.31 μV). The SPQ-based grouping showed a similar but slightly less pronounced trend. A repeated measures ANOVA with condition as a within-subjects factor and group (SPQ-based grouping) as a between-subjects factor revealed a significant main effect of condition, $F(4, 232) = 101.26$, $p < .001$, $\eta^{2g} = .36$, indicating that N1 amplitude differed significantly across the five experimental conditions. There was no significant main effect of group ($F(1, 58) = 0.92$, $p = .342$, $\eta^{2g} = .01$) and no condition × group interaction ($F(4, 232) = 0.62$, $p = .647$, $\eta^{2g} = .003$). Independent samples t-tests comparing control and high-schizotypy groups within each condition revealed no significant differences after Bonferroni correction for multiple comparisons (all $p > .05$). Using PDI as the grouping criterion, the repeated measures ANOVA showed a marginal main effect of group, $F(1, 58) = 3.41$, $p = .070$, $\eta^{2g} = .04$. The condition × group interaction remained non-significant, $F(4, 232) = 1.35$, $p = .254$, $\eta^{2g} = .008$. Post-hoc comparisons revealed different patterns compared to SPQ grouping. While no comparisons reached significance after Bonferroni correction, several showed notable trends: difference between control and high-schizotypy group in the mismatch condition approached significance. High-PDI participants consistently showed more negative N1 amplitudes across speaking conditions compared to controls, while listening conditions showed minimal group differences.

Supplementary Figure S8. ERP waveforms and N1 topographies for control and high-SPQ groups across conditions. (a–b) ERP waveforms for the veridical conditions (veridical_speak: purple, dashed vs. veridical_listen: teal, solid) are shown for the control group (a) and high-SPQ group (b).
(c–d) ERP waveforms for the stranger conditions (stranger_match: purple, dashed, stranger_mismatch: blue, dotted, and stranger_listen: brown, solid) are shown for the control group (c) and high-SPQ group (d).

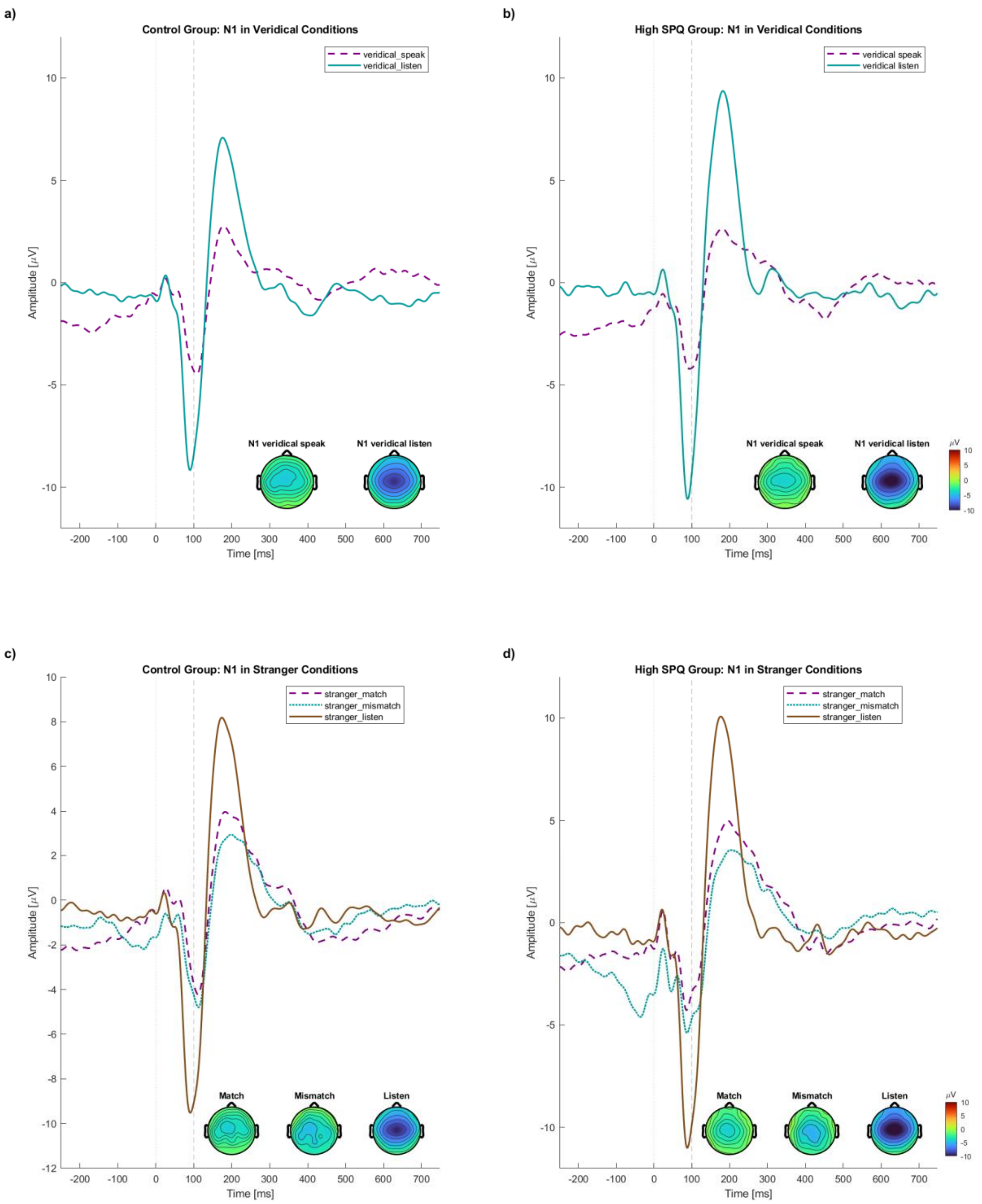

Supplementary Figure S9. ERP waveforms and N1 topographies for control and high-PDI groups across conditions. (a–b) ERP waveforms for the veridical conditions (veridical_speak: purple, dashed vs. veridical_listen: teal, solid) are shown for the control group (a) and high-PDI group (b).
(c–d) ERP waveforms for the stranger conditions (stranger_match: purple, dashed, stranger_mismatch: blue, dotted, and stranger_listen: brown, solid) are shown for the control group (c) and high-PDI group (d).

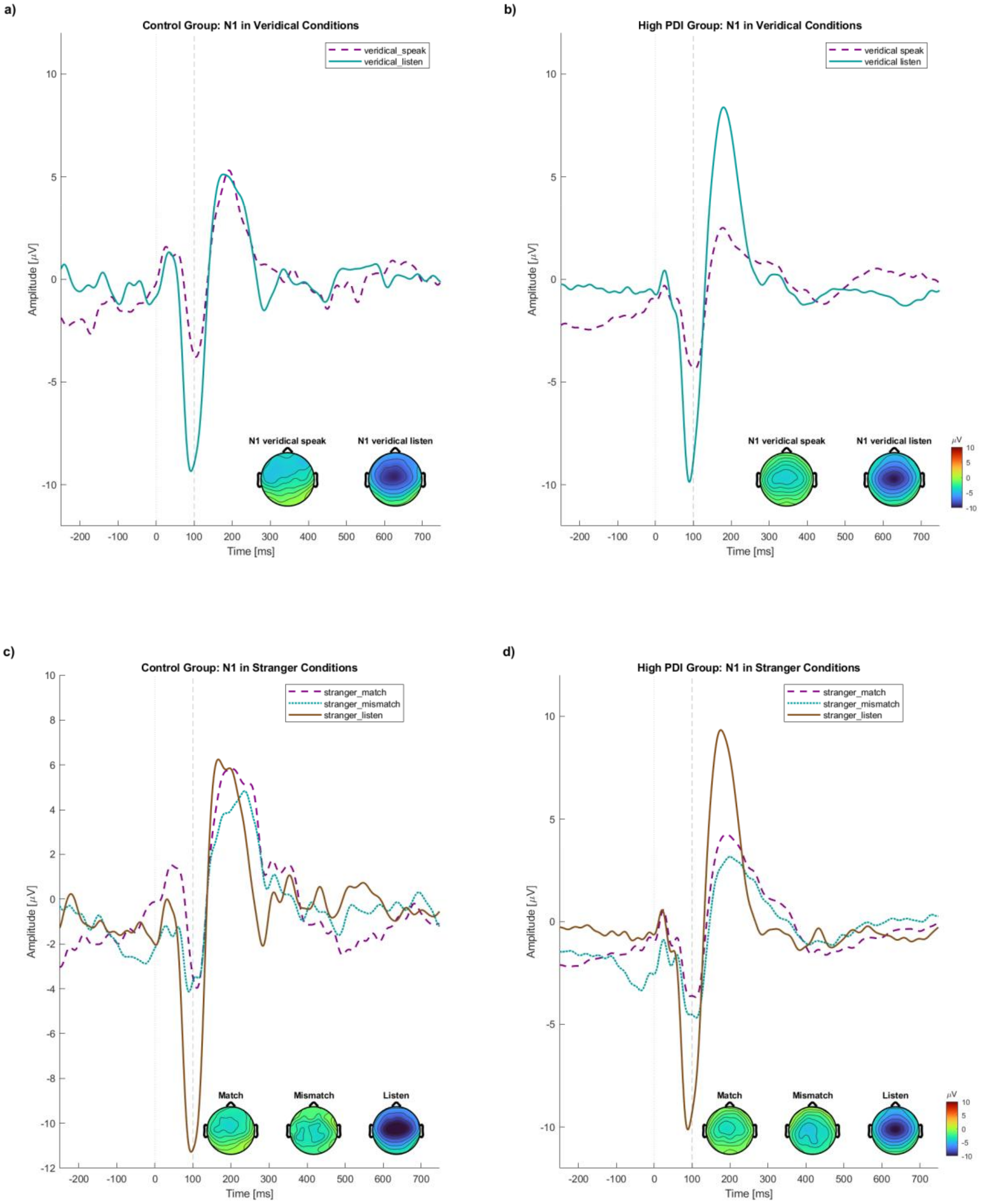

Supplement: Supplementary file 1 — Supplementary Material 1 [file 41598_2025_34714_MOESM1_ESM.pdf]
